# circFOXP1: a potential diagnostic and therapeutic target in human diseases

**DOI:** 10.3389/fimmu.2024.1489378

**Published:** 2024-11-13

**Authors:** Qiang Yi, Xinting Ouyang, Kui Zhong, Zheng Chen, Weijian Zhu, Gangfeng Zhu, Jinghua Zhong

**Affiliations:** ^1^ The First Clinical Medical College, Gannan Medical University, Ganzhou, Jiangxi, China; ^2^ Department of Oncology, The First Affiliated Hospital of Gannan Medical University, Ganzhou, Jiangxi, China

**Keywords:** circRNA, circFOXP1, molecular mechanisms, biomarker, treatment target

## Abstract

Circular RNA (circRNA) are a unique class of non-coding RNAs characterized by their covalently closed loop structures, which grant them properties such as stability and conservation. Among these, circFOXP1 has been implicated in various diseases, including cancers, respiratory, skeletal, and cardiovascular disorders. This review systematically examines circFOXP1’s role in disease progression, highlighting its involvement in critical biological processes, including cell proliferation, invasion, apoptosis, and autophagy. Mechanistically, circFOXP1 functions through miRNA sponging, protein interactions, and modulation of key signaling pathways such as Wnt and PI3K/AKT. We discuss its potential as a diagnostic and therapeutic target. Our analysis also identifies key unresolved questions, such as the precise regulatory networks involving circFOXP1 and its translation potential, offering pathways for future research.

## 1 Introduction

Circular RNAs (circRNA) are RNA molecules formed through an atypical splicing process, where the downstream 5′ splice site is joined to the upstream 3′ splice site, often involving exons, introns, or lariat introns (1). Back-splicing is an unconventional RNA splicing mechanism facilitated by the spliceosome, along with various cis- and trans-regulatory elements (2). The formation of circRNAs is tightly controlled by these factors (3). Cis-regulatory elements such as splice sites, enhancers, silencers, and especially elements near the junction sites, including inverted Alu repeats, play a key role in this process (4). CircRNAs were first visualized in the cytoplasm of eukaryotic cells using electron microscopy in 1979, with subsequent studies confirming their presence across higher eukaryotes (5, 6). Initially, circRNAs were considered rare byproducts of erroneous splicing (7). However, advancements in bioinformatics and high-throughput sequencing have now firmly established that circRNAs are broadly distributed in eukaryotic cells, where they play critical roles in gene expression regulation (8). To date, three principal types of circRNAs have been identified: exonic circRNA (9), circular intronic RNA (10), and exon-intron circRNA (11). Exonic circRNAs primarily function as miRNA sponges, sequestering miRNAs and thereby enhancing the expression of their target genes. Conversely, intron-containing circRNAs, including circular intronic RNAs and exon-intron circRNAs, predominantly localize to the nucleus, where they modulate the transcription of specific genes (12).

CircFOXP1, encoded by the FOXP1 gene on chromosome 3q13, generates multiple circular RNAs, with hsa_circ_0008234 (13), hsa_circ_0066523 (14), and hsa_circ_0001320 (15) being the most extensively studied. Recent research has increasingly highlighted the involvement of circFOXP1 in a range of human diseases, including malignancies (16), respiratory disorders (17), musculoskeletal conditions (18), reproductive system diseases (19), and cardiovascular ailments (20). As a circular RNA, circFOXP1 primarily functions as a miRNA sponge, regulating tumor-suppressive miRNAs such as miR-127-5p, miR-338-3p, and miR-547-5p, and influencing disease progression through protein interactions and modulation of signaling pathways like Wnt and PI3K/AKT. This highlights circFOXP1’s potential as a diagnostic and prognostic target in various pathologies.

FOXP1, a member of the FOX transcription factor family, is involved in gene regulation, cell growth, and differentiation (21, 22). Early studies revealed that FOXP1 plays a significant role in the development of the heart, lungs, brain, and B cells within the immune system (23–26). As research into FOXP1’s functions has progressed, its critical involvement in various cancers and non-neoplastic diseases has become increasingly apparent (27, 28). As research has progressed, the involvement of FOXP1, and by extension circFOXP1, in cancers and other diseases has become evident. This review explores the molecular mechanisms and biological significance of circFOXP1 and its potential as a biomarker and therapeutic target.

## 2 Production and subtypes of circRNA

**Figure 1:**
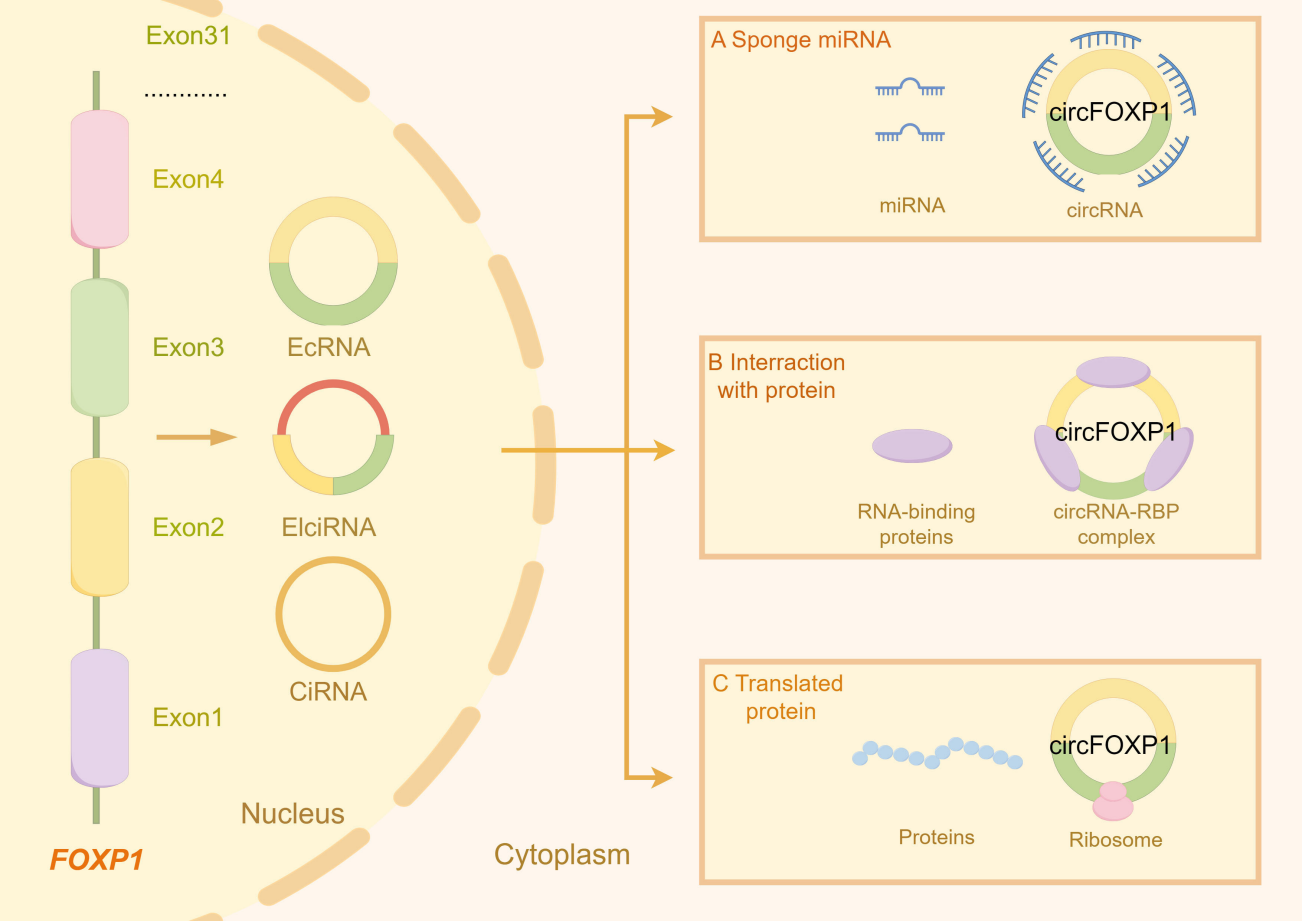
The figure illustrates the types of circRNA transcribed from the FOXP1 gene and the primary gene expression and functions associated with these circRNA molecules. **(A)** circFOXP1 acts as a miRNA sponge, binding to miRNAs and preventing them from interacting with their other targets. **(B)** circFOXP1 can also bind to RNA-binding proteins (RBPs) to form complexes. **(C)** circFOXP1 can be translated into proteins in the cytoplasm via ribosomes. The figure was generated using Figdraw (https://www.figdraw.com/static/index.html#/).

Circular RNAs (circRNA) are a distinct class of RNA molecules formed through atypical splicing events, where the 5′ and 3′ ends are covalently linked to create a closed circular structure, differentiating them from linear RNAs (29, 30). This unique circular configuration imparts greater stability to circRNAs and provides robust resistance to exonucleolytic degradation, thereby prolonging their persistence within the cell (31). The biogenesis of circRNAs involves two principal circularization mechanisms: exon circularization and intron circularization, resulting from the splicing of exonic or intronic sequences, respectively (32). Based on their origins and structural features, circRNAs can be classified into three main types: exonic circRNAs (EcircRNAs), circular intronic RNAs (ciRNAs), and exon-intron circRNAs (EIciRNAs) (33) (as shown in **Figure 1**). The formation of circRNAs is influenced not only by the splicing mechanism but also by spliceosome regulation, RNA-binding proteins, and the sequence characteristics of pre-mRNA (34, 35). As research advances, the critical roles of circRNAs in gene expression regulation (36), signal transduction (36), and cell cycle control (37) in disease development are increasingly recognized, highlighting circRNAs as pivotal regulatory molecules in cellular functions and pathophysiological processes rather than mere byproducts of genomic transcription.

## 3 Clinical features and *in vivo* model studies of circFOXP1 in human diseases

The clinical features and *in vivo* model studies of circFOXP1 in various human diseases are summarized in **Tables 1** and **2**.

**Table 1:** The clinicopathological characteristics of circFOXP1 in various diseases.

Disease type	Number of cases	Expression	Clinicopathological characteristics	Prognosis	Refs.
Cutaneous squamous cell carcinoma (cSCC)	10	high	/	poor	(13)
Colorectal cancer (CRC)	78	high	tumor stage and overall survival (OS)	poor	(16)
Lung adenocarcinoma (LUAD)	50	low	tumor size and survival	poor	(38)
	153	high	OS	poor	(39)
	15	high	/	poor	(40)
Gallbladder cancer (GBC)	40	high	lymph node metastasis, TNM (Tumor-Node-Metastasis) stage	poor	(41)
Non-small cell lung cancer (NSCLC)	105	high	T stage and lymphatic metastasis	poor	(42)
Gastric cancer (GC)	56	high	tumor size, lymph node metastasis, TNM stage and survival	poor	(43)
Osteosarcoma	40	high	OS	poor	(44)
Epithelial ovarian cancer (EOC)	200	high	FIGO(International Federation of Gynecology and Obstetrics) stage, primary tumor size, lymph node metastasis, distant metastasis, residual tumor diameter, and clinical response	poor	(45)
Intrahepatic cholangiocarcinoma (ICC)	537	low	survival, recurrence rates	poor	(46)
Hepatocellular carcinoma (HCC)	93	high	larger tumor size, microvascular invasion, advanced TNM stage, and predicted poor prognosis	poor	(47)
Renal cell carcinoma (RCC)	30	high	/	poor	(48)
acute ische-mic stroke (AIS)	288	low	cerebral infarct volume, poor prognosis, fibrinogen and D-dimer contents	poor	(49)
Recurrent pregnancy loss (RPL)	56	low	Number of pregnancy loss	poor	(19)
Keloid	70	high	/	poor	(50)
Osteoporosis (OP)	40	low	/	poor	(18)

“/”, absence of relevant studies or data.

**Table 2:** Effects of circFOXP1 on growth and metastasis of cancer xenografts.

Disease type	Animal models	Results	Refs.
CRC	5-week-old nude male BALB/c mice	Δ circFOXP1: ↓ proliferation, metastatic, ki67	(51)
	2-month age male BALB/c nude mice	Δ circFOXP1: ↓ proliferation, ki67 ↑drug-sensitizing	(16)
LUAD	female nude mice	↑circFOXP1: ↓ tumor growth, ki67 ↑cleavage caspase 3	(38)
	6-week-old SCID/nude mice	Δ circFOXP1: ↓ tumor growth, ki67	(39)
GBC	3-week-old male nude mice	↑circFOXP1: ↑tumor growth, ki67;Δ circFOXP1: ↓ tumor growth, ki67	(41)
GC	3-week-old BALB/c nude mice	Δ circFOXP1: ↓ tumor growth	(43)
Osteosarcoma	6-week-old BALB/c nude mice	Δ circFOXP1: ↓ tumor volume, CD31 and microvascular density	(44)
EOC	nude mice	Δ circFOXP1: ↓tumor growth ↑ sensitivity of DDP(cisplatin)	(45)
ICC	nude mice	↑circFOXP1: ↓ tumor growth, ki67; Δ circFOXP1: ↑tumor growth, ki67	(46)
HCC	5-week-old male BALB/c nude mice	Δ circFOXP1: ↓tumor volume and weight	(47)
AIS	Male C57BL/6JNifdc mice	↑circFOXP1: ↓cerebral infarct volume	(49)
Pulmonary fibrosis(PF)	8-week-old C57BL/6 mice	Δ circFOXP1: ↓Pulmonary Fibrosis	(17)
Keloid	6-week-old male BALB/C nude mice	↑circFOXP1: ↑ keloid, ki67	(50)
Atherosclerosis (AS)	8-week-old apolipoprotein E-deficient (ApoE−/−) mice	Δ circFOXP1: ↑TNF-α,IL-6, IL-1β	(20)
OP	6-week-old female BALB/C homozygous nude (nu/nu) mice	Δ circFOXP1: ↓newly constructed bone, collagen fibre bundle, brown stained granule	(18)

Δ, knock-down or deletion. ↑ indicates overexpression, an increase, or an upward trend, while the symbol ↓ signifies a decrease or a downward trend.

### 3.1 CircFOXP1 in clinical research

CircRNAs, primarily non-coding RNAs generated through back-splicing, have garnered significant interest due to their crucial roles in various diseases. While most circRNAs do not code for proteins, some exhibit potential protein-coding capabilities. CircFOXP1, in particular, shows markedly dysregulated expression across a wide range of conditions, including malignancies, respiratory disorders, musculoskeletal diseases, reproductive system disorders, and cardiovascular diseases (as shown in **Table 1**). These expression patterns correlate with diverse clinical features, highlighting circFOXP1’s potential as a key biomarker in these pathologies.

### 3.2 Animal studies related to circFOXP1

### 3.3 CircFOXP1 in malignant tumors

CircFOXP1 has been extensively studied in the context of malignant tumors, including lung cancer, gastric cancer, liver cancer, and colorectal cancer, all of which are associated with high incidence and mortality rates. The poor prognosis for these cancers is largely attributed to the lack of effective early diagnostic methods and therapeutic targets.

#### 3.3.1 Colorectal cancer

Colorectal cancer (CRC) ranks as the third most common cancer and the second leading cause of cancer-related mortality worldwide (52, 53). Despite its high global death rate, CRC is preventable and curable with timely intervention (54, 55). Increasing evidence indicates that circFOXP1 is significantly upregulated in colon cancer cells and tissues compared to normal tissues (51). These findings suggest that circFOXP1 may play a crucial role in the initiation and progression of colon cancer. *In vivo* studies have demonstrated that circFOXP1 promotes tumor growth, metastasis, invasion, and reduces sensitivity to chemotherapy. Furthermore, the expression level of circFOXP1 correlates with tumor stage and overall survival, with high circFOXP1 expression associated with advanced tumor stages and poorer survival outcomes (16). While research on circFOXP1 in colon cancer is substantial, studies in rectal cancer are limited, necessitating further investigation to clarify its role in rectal cancer. Nevertheless, circFOXP1 remains a promising candidate for diagnostic, therapeutic, and prognostic applications in colorectal cancer.

#### 3.3.2 Lung cancer

Lung cancer is primarily classified into small cell lung cancer (SCLC) and non-small cell lung cancer (NSCLC) (56). Among NSCLC, lung adenocarcinoma (LUAD) is a prominent subtype (57). The role of circFOXP1 in lung adenocarcinoma exhibits a dual nature. On one hand, some studies have reported that circFOXP1 is downregulated in LUAD tissues, where it can inhibit tumor cell growth and accelerate apoptosis both *in vivo* and *in vitro* (38). On the other hand, elevated levels of circFOXP1 have been observed in lung adenocarcinoma cells and tissues compared to normal tissues, with high circFOXP1 expression correlating with poor patient prognosis (39, 40). Additionally, in some bioinformatics studies, circFOXP1 has been identified as a tumor suppressor molecule in lung cancer (58, 59). This dichotomy suggests that circFOXP1 may exert different biological functions depending on the molecular environment or experimental conditions. This highlights the need for more in-depth research to elucidate the specific mechanisms of circFOXP1 in various contexts and its potential as a therapeutic target. Investigating these contrasting findings could enhance our understanding of circFOXP1’s multifaceted role in lung cancer and inform the development of personalized treatment strategies. However, research on circFOXP1 in the highly malignant SCLC remains limited.

#### 3.3.3 Gastric cancer

Gastric cancer (GC) ranks as the fifth most common cancer globally and the third leading cause of cancer-related mortality (60, 61). The high mortality rate is largely due to the lack of early clinical symptoms and diagnostic tools, resulting in most GC patients being diagnosed at an advanced stage (62). Consequently, the prognosis for GC patients is generally poor (63). Studies have reported that elevated circFOXP1 expression in gastric cancer tissues and cells is closely associated with unfavorable clinical outcomes, while patients with lower circFOXP1 levels tend to have better prognoses. This is often reflected in clinical features such as lymphatic metastasis, TNM stage, and tumor size. Moreover, patients with high circFOXP1 levels exhibit significantly higher overall mortality compared to those with lower levels. *In vivo* experiments further demonstrate that knocking down circFOXP1 inhibits GC tumor growth (43). Collectively, these findings underscore the critical role of circFOXP1 in GC progression and highlight its potential clinical value as a prognostic marker and therapeutic target in gastric cancer.

#### 3.3.4 Liver cancer

Liver cancer is the sixth most common cancer globally and the third leading cause of cancer-related deaths (52, 64). Hepatocellular carcinoma (HCC) is the most prevalent form of primary liver cancer (65). Extensive research has demonstrated a close association between circRNA expression and HCC (66, 67). In studies focusing on hepatocellular carcinoma with Catenin beta 1 (CTNNB1) mutations, circFOXP1 expression was found to be dysregulated in liver cancer tissues (68). Additionally, research by Wang et al. (47) revealed that high circFOXP1 expression is significantly correlated with larger tumor size, microvascular invasion, advanced TNM stage, and poor prognosis. In serological studies, the diagnostic ROC curve of circFOXP1 suggests its potential value as a biomarker in HCC. *In vitro* experiments indicate that knocking down circFOXP1 inhibits HCC cell proliferation and invasion while promoting apoptosis; *in vivo*, circFOXP1 exacerbates HCC tumor formation and increases Ki67 expression. These findings suggest that circFOXP1 accelerates HCC progression. Intrahepatic cholangiocarcinoma (ICC), though relatively rare, is an extremely aggressive type of tumor that responds poorly to chemotherapy and immunotherapy (69). ICC accounts for approximately 10%-15% of all liver cancer cases (70). Interestingly, research on circFOXP1 in ICC contrasts with findings in HCC. Overexpression of circFOXP1 in ICC reduces tumor size and lowers Ki67 expression, while knockdown of circFOXP1 produces the opposite effect. High circFOXP1 expression is associated with longer survival times and lower recurrence rates in ICC patients (46). These results indicate that circFOXP1 plays an antitumor role in ICC and may serve as a favorable prognostic marker. In summary, circFOXP1 exhibits distinct mechanisms of action in HCC and ICC. This differential role suggests that circFOXP1 may function through different molecular pathways in various types of liver cancer, necessitating divergent therapeutic strategies for HCC and ICC. These findings provide valuable insights for further exploration of the molecular mechanisms of circFOXP1 in different liver cancers.

#### 3.3.5 Gallbladder cancer

Gallbladder cancer (GBC) is a relatively rare but highly lethal malignancy with a poor prognosis (71, 72). Radical surgery is the only curative treatment for GBC, but the high risk associated with late-stage surgery limits its applicability (73). Therefore, novel immunotherapies for advanced GBC and early diagnostic methods are of paramount importance. Research has shown that high circFOXP1 expression is closely associated with lymph node metastasis and advanced TNM stage in GBC patients. The expression levels of circFOXP1 in GBC tissues are significantly higher than in normal, non-tumorous gallbladder tissues. Downregulation of circFOXP1 impairs GBC cell proliferation, induces G1-S phase arrest, and increases apoptosis rates, with concomitant reductions in the expression of PCNA, MMP9, and AKT. Conversely, upregulation of circFOXP1 enhances GBC cell proliferation. Moreover, in animal model experiments, circFOXP1 knockdown inhibited tumor growth, while its overexpression had the opposite effect (41). Thus, elevated circFOXP1 levels significantly promote GBC cell proliferation, invasion, and distant metastasis. These findings suggest that circFOXP1 could serve as a novel diagnostic biomarker and potential therapeutic target for GBC.

#### 3.3.6 Osteosarcoma

Osteosarcoma is one of the most common primary bone tumors, with a 5-year survival rate of less than 20% after metastasis (74). The mechanisms of neovascularization include angiogenesis, sprouting, intussusception, vessel co-option, vasculogenic mimicry, and lymphangiogenesis (75). Angiogenesis provides cancer cells with oxygen and nutrients, playing a crucial role in cancer cell survival and metastasis (76). Recent studies have indicated that circFOXP1 is highly expressed in osteosarcoma and is negatively correlated with patient survival rates. Functional mouse models have demonstrated that low expression of circFOXP1 reduces microvascular density in osteosarcoma tumors, leading to a decrease in tube formation ability (44). These findings suggest that circFOXP1 promotes neovascularization in osteosarcoma and plays a critical role in angiogenesis. Although progress has been made in osteosarcoma treatment, patient outcomes remain poor. Therefore, exploring new therapeutic strategies targeting neovascularization in osteosarcoma is of significant importance.

#### 3.3.7 Other malignant tumors

Ovarian cancer is the eighth most common cancer in women and is known for its high malignancy (77, 78). CircRNAs play a major role in the proliferation, migration, and progression of ovarian cancer cells (79). Reports have shown that the expression of exosomal circFOXP1 is significantly higher in patients with epithelial ovarian cancer (EOC) who are resistant to cisplatin (DDP) compared to DDP-sensitive patients. Additionally, the expression of exosomal circFOXP1 in patient serum is significantly correlated with FIGO stage, primary tumor size, lymph node metastasis, distant metastasis, residual tumor diameter, and clinical response. Overexpression of circFOXP1 is associated with poor prognosis in EOC patients. Moreover, knocking down circFOXP1 enhances the sensitivity of EOC cells to DDP and reduces the expression of the tumor growth marker Ki67 (45). Renal cell carcinoma (RCC) is the second most common cancer of the urinary system (80). Studies have found that downregulating circFOXP1 can inhibit RCC proliferation, migration, invasion, and reduce the Warburg effect (48). In cutaneous squamous cell carcinoma (cSCC), circFOXP1 expression is upregulated compared to non-cancerous skin tissues. Silencing circFOXP1 has been shown to inhibit the proliferation of cSCC cells (13). Therefore, circFOXP1 may serve as a potential therapeutic target in these cancers. Targeting circFOXP1 could help suppress tumor progression and overcome chemotherapy resistance.

### 3.4 circFOXP1 in non-malignant diseases

#### 3.4.1 Osteonecrosis of the femoral head

Osteonecrosis of the femoral head (ONFH) is a common and challenging orthopedic condition, often leading to hip pain and functional impairment. It has a high disability rate, imposing a heavy burden on patients, their families, and society (81). Studies have shown that circFOXP1 is upregulated during the osteogenic induction of bone marrow mesenchymal stem cells (BMSCs). Knocking down circFOXP1 can inhibit the proliferation and osteogenic differentiation of BMSCs, with a noted decrease in the expression levels of RUNX2, OPN, and OCN (14). CircFOXP1 is thus considered a key activator in the treatment of ONFH.

#### 3.4.2 Acute ischemic stroke

Acute ischemic stroke (AIS) is a leading cause of death worldwide, with high rates of disability and mortality (82, 83). Elevated circFOXP1 expression is considered a protective factor for AIS prognosis, as indicated by both univariate and multivariate analyses. ROC curve analysis has demonstrated the potential of circFOXP1 as a predictor of poor outcomes. Studies have shown that circFOXP1 expression is significantly reduced in the peripheral blood of AIS patients and is associated with the severity and prognosis of AIS. Specifically, circFOXP1 levels are inversely correlated with infarct size and the levels of fibrinogen, D-dimer, α-hydroxybutyrate dehydrogenase, and lactate dehydrogenase in the blood. This trend is also observed in animal models. Knockdown of circFOXP1 induces the expression of pro-apoptotic proteins (Bax and Caspase-3) and suppresses the expression of the anti-apoptotic protein Bcl2 in human glioblastoma cells. Conversely, overexpression of circFOXP1 inhibits Bax and Caspase-3 expression while inducing Bcl2 expression (49). CircFOXP1 holds potential as a biomarker for assessing prognosis in AIS patients and may serve as a novel therapeutic target for treating AIS.

#### 3.4.3 Pulmonary fibrosis

Pulmonary fibrosis (PF) gradually affects the lung parenchyma, leading to impaired gas exchange, difficulty breathing, decreased quality of life (QoL), and ultimately resulting in respiratory failure and death (84). In studies investigating the effects of human umbilical cord mesenchymal stem cells (hucMSCs) on PF, it has been found that the therapeutic efficacy of hucMSCs in inhibiting PF is dependent on the downregulation of circFOXP1. In animal experiments, mice treated with hucMSCs exhibited significant improvements, including thinner alveolar walls, better-preserved alveolar structure, reduced alveolar inflammation, and decreased collagen deposition. Furthermore, fibrosis-related proteins such as vimentin, α-SMA, collagen I, collagen III, and the differentiation marker S100A4 were significantly reduced in the hucMSCs-treated group (17). These findings suggest that circFOXP1 plays a promotive role in the development of PF, and that hucMSCs may offer a promising therapeutic approach by downregulating circFOXP1.

#### 3.4.4 Recurrent pregnancy loss

Recurrent pregnancy loss (RPL) is defined as the failure of two or more clinical pregnancies and is a common issue among women of reproductive age (85, 86). Clinical investigations have revealed that the level of miR-143-3p is significantly upregulated in the placental tissues of RPL patients, while circFOXP1 acts as a ceRNA for miR-143-3p in trophoblast cells. It has been observed that the expression of circFOXP1 is downregulated in these patients. Overexpression of circFOXP1 has been shown to enhance the viability of HTR8/SVneo cells, reduce apoptosis, and promote cell migration and invasion, thereby improving trophoblast cell function (19). The downregulation of circFOXP1 may lead to trophoblast dysfunction, thereby increasing the risk of RPL. Therefore, circFOXP1 could be a potential therapeutic target for RPL.

#### 3.4.5 Keloids

Keloids are pathological scars that cause significant functional and cosmetic burdens (87). A study found that the levels of circFOXP1 were higher in keloid tissues compared to normal skin tissues. Overexpression of circFOXP1 significantly promoted the proliferation and migration of human skin fibroblasts (HSFs) while inhibiting apoptosis. This upregulation of circFOXP1 led to enhanced keloid growth *in vivo*, along with increased deposition of extracellular matrix (ECM) proteins such as α-SMA, collagen I, and collagen III (50). Thus, circFOXP1 plays a promotive role in keloid formation by accelerating fibroblast proliferation and migration, as well as increasing ECM protein deposition, thereby driving the development of keloids.

#### 3.4.6 Atherosclerosis

Atherosclerosis is a chronic inflammatory vascular disease driven by both traditional and non-traditional risk factors (88). Research has shown that circFOXP1 is significantly upregulated in atherosclerosis (AS) mouse models and AS cell models. In addition, in apolipoprotein E-deficient mice induced by a high-fat diet (HFD), circFOXP1 was found to promote the upregulation of pro-inflammatory cytokines such as interleukin IL-6, TNF-α, and IL-1β, further exacerbating endothelial cell injury (20). CircFOXP1 plays a promotive role in the development of atherosclerosis, with its upregulation associated with increased inflammatory factors, thereby worsening endothelial cell damage. This suggests that circFOXP1 may be an important regulator of inflammation in atherosclerosis and could serve as a potential therapeutic target.

#### 3.4.7 Osteoporosis

Osteoporosis (OP) is a skeletal disorder characterized by compromised bone structure and strength, leading to an increased risk of fragility fractures (89). CircFOXP1 has been found to be significantly downregulated in the bone tissue of OP patients. Furthermore, circFOXP1 promotes new bone formation in mice, with more collagen fiber bundles and an increased presence of brown-staining particles, indicating its role in regulating osteogenesis in human adipose-derived mesenchymal stem cells (hASCs) (18). However, the clinical trial involving the isolation of hASCs from patients, seeding them onto composite grafts, and reimplanting them into fracture sites (https://clinicaltrials.gov/study/NCT01532076) has yet to be completed. Therefore, further preclinical and clinical studies are necessary to determine the therapeutic potential of circFOXP1 in treating osteoporosis.

#### 3.4.8 Mesenchymal stem cell lineage specification

CircFOXP1 is recognized as a specific molecular marker of undifferentiated mesenchymal stem cells (MSCs). Knockdown of circFOXP1 has been shown to reduce MSC growth, with a concomitant decrease in the expression levels of MSC markers such as CD164, PDPN, CD146, and GLI1. Additionally, the abundance of CD90+/CD146+ and CD73+/CD105+ double-positive populations diminishes, leading to impaired MSC differentiation capabilities (90). Therefore, circFOXP1 plays a crucial role in maintaining the undifferentiated state, marker expression, and differentiation potential of MSCs. CircFOXP1 may be a key molecule in regulating the biological functions of MSCs.

## 4 Regulatory molecular mechanisms of circFOXP1 in human diseases

**Figure 2:**
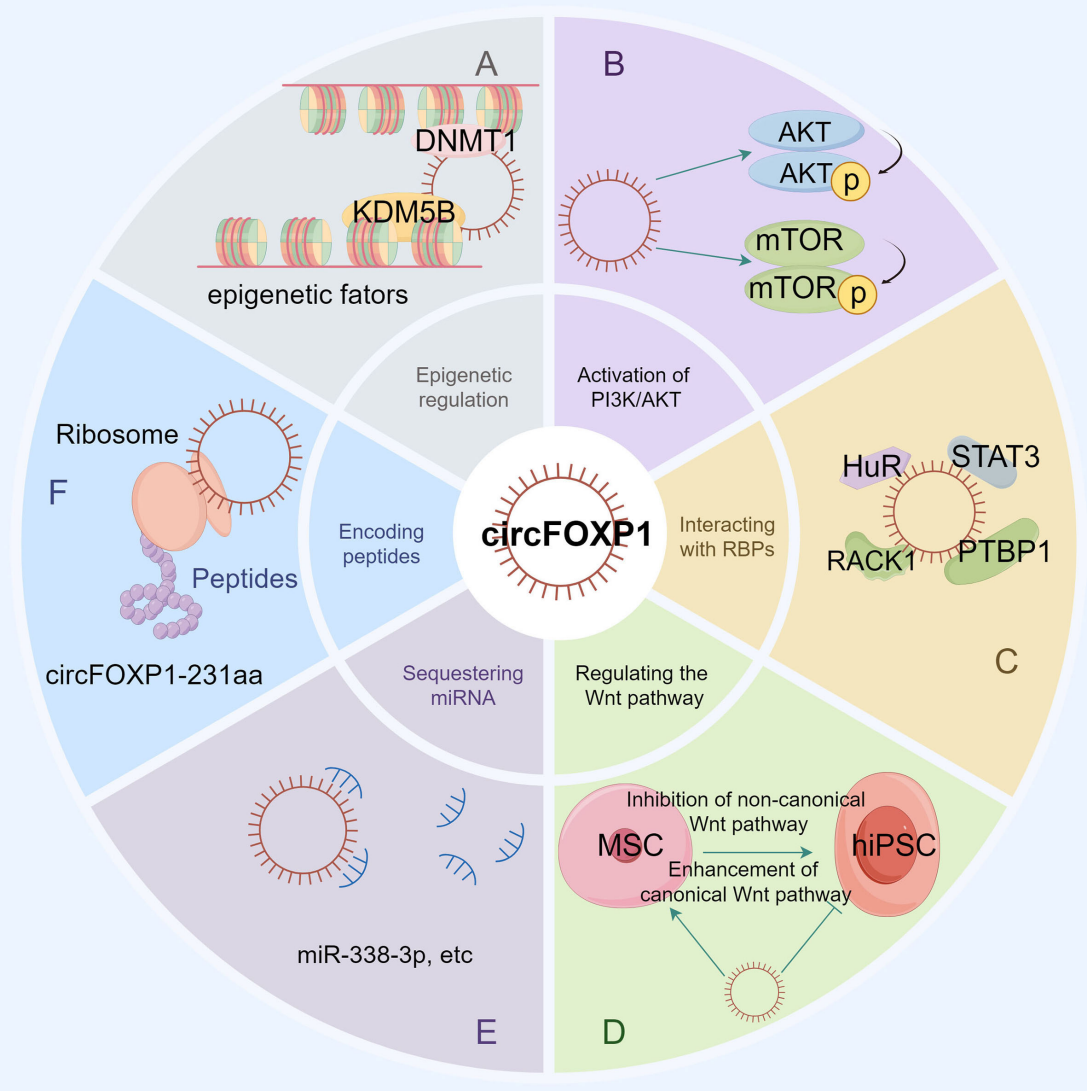
The figure illustrates the various functions and molecular interactions of circFOXP1 in cellular processes. **(A)** Epigenetic regulation, showing how circFOXP1 influences DNA methylation and histone modification by recruiting DNMT1 and KDM5B. **(B)** The role of circFOXP1 in the PI3K/AKT signaling pathway. **(C)** Sequestration of RNA-binding proteins, demonstrating how circFOXP1 functions by binding to RNA-binding proteins. **(D)** The involvement of circFOXP1 in both canonical and non-canonical Wnt signaling pathways. **(E)** The miRNA sponge function of circFOXP1. **(F)** The translation of circFOXP1 into the protein circFOXP1-231aa via ribosomes. (by Figdraw2.0).

CircFOXP1 regulates gene expression involved in the development of various diseases through multiple mechanisms. These include acting as a miRNA sponge, enEMTgaging in RNA-binding protein mechanisms, serving as a transcriptional regulator, and directly encoding proteins. Additionally, circFOXP1 indirectly influences both classical and non-classical signaling pathways (as shown in **Figures 2B, D**). CircFOXP1 itself is also subject to regulation by upstream transcription factors and other related mechanisms. The regulatory molecular mechanisms of circFOXP1 in various human diseases are detailed in **Table 3**.

**Table 3:** The biological functions and mechanisms of circFOXP1 in diseases.

Disease type	Assessed Cell Lines	Expression	Functional	Related gene	Refs.
cSCC	A431	Upregulated	Promote proliferation	miR−127−5p/ADCY7	(13)
CRC	HCT116, SW480, HCT8, and DLD-1	Upregulated	Promote proliferation, migration, and invasion	miR-338-3p/ETS1/PI3K/AKT/mTOR	(51)
	LOVO、HCT116、SW480、SW620	Upregulated	Promote proliferation,	DNMT1/FOXP1	(16)
LUAD	A549, SPCA1, H1299, and PC9	Downregulated	Inhibit proliferation and promote apoptosis	miR-574-5p/RND3	(38)
	/	Upregulated	/	miR-490-3p/SYT1	(91)
	/	Upregulated	Promote proliferation, migration, and invasion	miR-520a-5p/SLC7A11	(39)
	A549, H1299	Upregulated	Promote proliferation and inhibit apoptosis	miR-185-5p/WNT1	(40)
	A549	Upregulated	/	/	(15)
GBC	NOZ, GBC-SD, EHGB-1, SGC-996 and OCUG-1	Upregulated	Promote proliferation, migration, and invasion, inhibit apoptosis	circFOXP1/PTBP1 miR-370/PKLR	(41)
NSCLC	/	Upregulated	/	miR-370-3p or miR-18a-5p	(42)
GC	HGC-27, MKN-45, MKN-28, and SGC-7901	Upregulated	Promote proliferation and invasion	ALKBH5/circFOXP1/miR-338-3p/SOX4	(43)
Osteosarcoma	U2OS, MG-63, HOS, and SAOS-02	Upregulated	angiogenesis	miR-127-5p/CDKN2AIP	(44)
EOC	COC1, OVCAR3, SKOV3, SKOV3/DDP	Upregulated	Promote proliferation, sensitivity to DDP and paclitaxel	miR-22/CEBPG and miR-150-3p/FMNL3	(45)
ICC	CCLP1, SG231, HCCC-9810 and RBE	Downregulated	Inhibit proliferation and invasion	OTUD4/NCOA4	(46)
HCC	SNU-387, HepG2, Hep3B, Huh7, SMMC-7721, HCCLM3	Upregulated	Promote proliferation, invasion, inhibit apoptosis	SOX9/circ-FOXP1/miR-875-3p or miR-421	(47)
RCC	ACHN, 786-O, OSRC-2, A 498, and CAKI-1	Upregulated	Promote proliferation, migration, invasion, and Warburg Effect	ZNF263/circFOXP1/miR-423-5p/U2AF2	(48)
Osteonecrosis of the femoral head (ONFH)	BMSC	Downregulated	Inhibit proliferation and osteogenic differentiation	KDM5B/PTEN/PI3K/AKT	(14)
AIS	A172	Downregulated	Inhibit apoptotic	QKI/circFOXP1/STAT3	(49)
PF	hucMSCs	Upregulated	Promote proliferation, migration	HuR-EZH2/STAT1/FOXK1	(17)
RPL	HTR8/SVneo	Downregulated	Inhibits proliferation, migration, invasion and EMT, promote apoptosis	miR–143–3p/S100A11	(19)
Keloid	HSF	Upregulated	Promote proliferation, migration, and inhibit apoptosis	RACK1	(50)
AS	HUVECs	Downregulated	Inhibit proliferation and promote apoptosis	miR-185-5p/BCL-2	(20)
OP	hASC	Downregulated	Inhibit osteogenic differentiation	miR-33a-5p/FOXP1	(18)

“/”, absence of relevant studies or data, but in mechanistic studies “/”, representing regulatory or interaction relationships between molecules.

### 4.1 Downstream mechanisms

#### 4.1.1 Competitive endogenous RNA sponging activity

**Figure 3:**
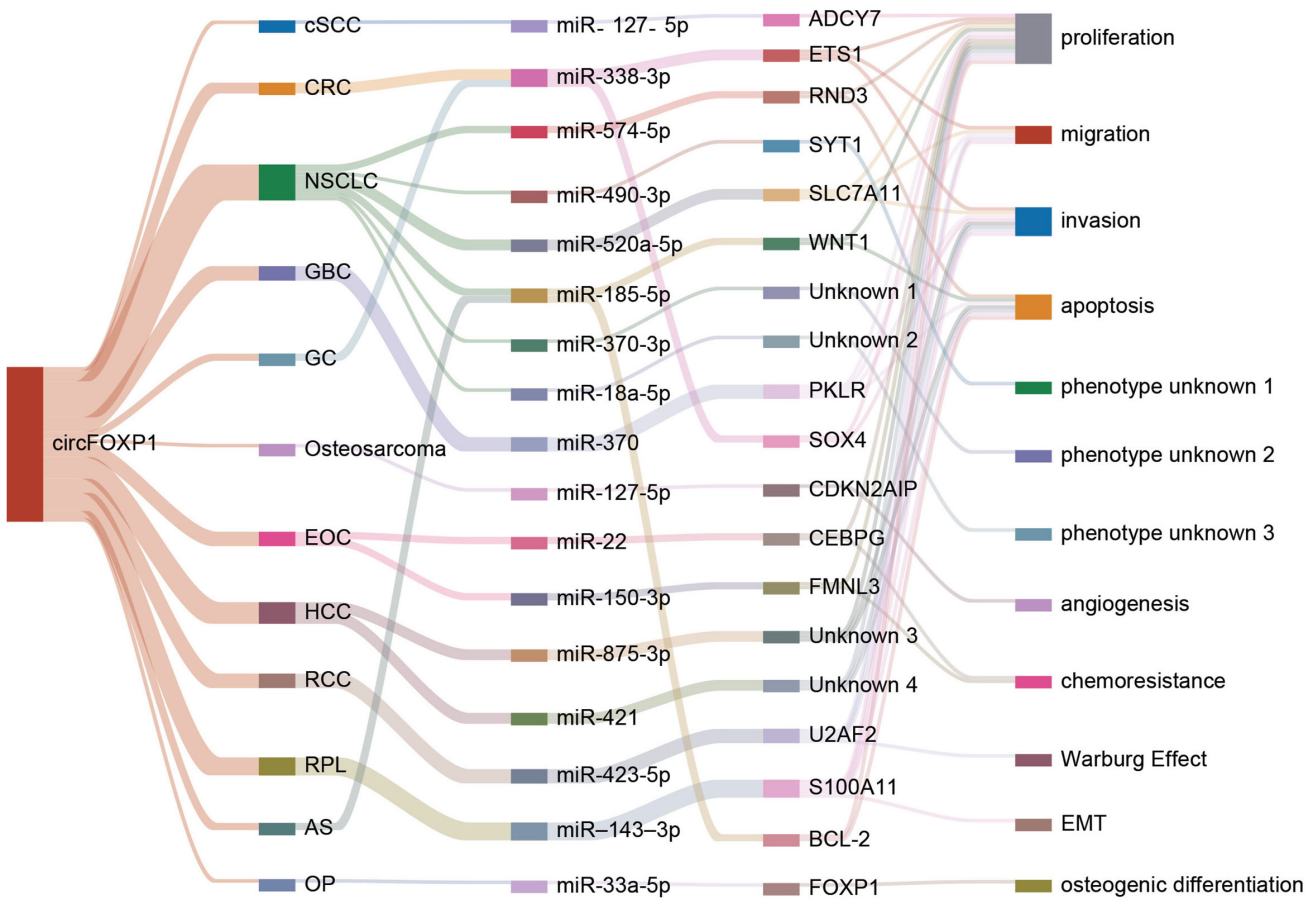
The figure illustrates how circFOXP1 regulates downstream target genes by sponging different miRNAs and highlights its interactions and functional effects across various cancer types and cellular processes. cSCC, Cutaneous Squamous Cell Carcinoma; CRC, Colorectal Cancer; NSCLC, Non-Small Cell Lung Cancer; GBC, Gallbladder Cancer; GC, Gastric Cancer; Osteosarcoma, Osteosarcoma; ECO, Esophageal Cancer; HCC, Hepatocellular Carcinoma; RCC, Renal Cell Carcinoma; RPL, Retroperitoneal Liposarcoma; AS, Angiosarcoma; OP, Ovarian Cancer. The various colored lines represent distinct molecules or traits, highlighting circFOXP1’s interactions and functional effects across different cancer types and cellular processes.

The competitive endogenous RNA (ceRNA) regulatory network represents a pivotal mechanism through which circFOXP1 exerts its influence in disease pathogenesis. By functioning as a ceRNA, circFOXP1 can act as a miRNA sponge, thereby modulating the expression of downstream messenger RNAs (mRNAs) and influencing disease progression (as shown in **Figure 3** and **Figure 2E**). Theoretically, any RNA possessing miRNA response elements (MREs) has the potential to bind miRNAs and thus perform the role of a ceRNA (92). Given their inherent MREs, circRNAs naturally possess the capability to sequester miRNAs (93). Notably, circFOXP1 has been identified to sponge miR-127-5p, thereby regulating ADCY7 and CDKN2AIP, which promotes the progression and angiogenesis of epithelial ovarian cancer and osteosarcoma, respectively (13, 44). Similarly, a series of experimental analyses in colorectal cancer cell lines have confirmed the existence of the circFOXP1/miR-338-3p/ETS1 axis (51). Interestingly, circFOXP1 exhibits a dual role in lung cancer, acting as both an oncogenic factor and a tumor suppressor. On one hand, circFOXP1 can sequester miR-490-3p, miR-520a-5p, miR-185-5p, miR-370-3p, or miR-18a-5p, preventing the degradation of their downstream target transcripts, thereby enhancing the expression of SYT1, SLC7A11, and WNT1, which in turn promotes tumor cell proliferation, migration, and invasion (39, 40, 42, 91). On the other hand, the circFOXP1/miR-574-5p/RND3 axis has been found to inhibit cell proliferation and promote apoptosis (38). Moreover, circFOXP1 can regulate various axes, including miR-338-3p/SOX4 (43), miR-150-3p/FMNL3 (45), miR-423-5p/U2AF2 (48), miR–143–3p/S100A11 (19), miR-185-5p/BCL-2 (20), and miR-33a-5p/FOXP1 (18), thereby playing a critical role in disease initiation, progression, and response to therapy by modulating processes such as cell proliferation, migration, invasion, apoptosis, drug resistance, epithelial-mesenchymal transition (EMT), and the Warburg effect.In summary, circFOXP1, functioning as a ceRNA, intricately modulates multiple biological properties of human diseases through its interactions with miRNAs. This mechanism is not only fundamental to the biological behavior of tumor cells but also significantly impacts disease progression and prognosis.

#### 4.1.2 Interactions with RNA-binding proteins

Beyond its role as a miRNA sponge, circRNA can also function as a sponge for RNA-binding proteins (RBPs). For instance, Zhang et al. identified RACK1, an RNA-binding protein, as a potential target of circFoxp1 through RNA pull-down assays. RACK1 is known to inhibit collagen synthesis in keloid fibroblasts by suppressing the TGF-β1/Smad signaling pathway (94). CircFoxp1 can downregulate RACK1 expression, potentially serving as a modulator of cell proliferation and apoptosis throughout the keloid formation process (50). In studies on pulmonary fibrosis, the inhibitory effects of hucMSC treatment on lung fibrosis were found to be dependent on the downregulation of circFOXP1. hucMSC treatment promotes the circFOXP1-mediated autophagy process by blocking the translocation of human antigen R (HuR) and facilitating its degradation, leading to a significant reduction in autophagy-negative regulators EZH2, STAT1, and FOXK1, thereby ameliorating lung mononuclear fibrosis (17). Additionally, under hypoxic conditions, the expression of circFOXP1 in the brain is reduced. This reduction exacerbates brain injury following ischemia by binding to and enhancing the ubiquitination of STAT3, accelerating the degradation of signal transducer and activator of transcription 3 (STAT3) protein, ultimately triggering apoptotic signaling pathways (49). Furthermore, Wang et al. discovered that the RNA-binding protein PTBP1 interacts with circFOXP1 in GBC cells. The exogenous expression of circFOXP1 enhances PTBP1 expression by increasing its translocation from the nucleus to the cytoplasm, while knockdown of circFOXP1 results in the retention of PTBP1 within the nucleus (41). Through its interactions with various RNA-binding proteins, circFOXP1 plays a regulatory role in numerous diseases (as shown in **Figure 2C**). These interactions not only affect key biological processes such as cell proliferation, apoptosis, and autophagy but also have significant regulatory functions in specific diseases like keloid, pulmonary fibrosis, cerebral ischemia, and gallbladder cancer, suggesting that circFOXP1 could be an important regulatory factor in these conditions.

#### 4.1.3 Epigenetic mechanisms

Epigenetics refers to changes in gene expression that are not attributable to alterations in the DNA sequence itself but rather to chemical modifications of DNA and associated proteins. These modifications can profoundly influence gene expression, cellular differentiation, tissue development, and susceptibility to diseases (95, 96). Among the various epigenetic mechanisms, DNA methylation is particularly notable for its extensive role in gene expression regulation and developmental processes (97, 98). The epigenetic mechanisms associated with circFoxp1 are also integral to its interactions with RBPs (**Figure 2A**). Research in colorectal cancer has revealed that overexpression of circFoxp1 significantly increases the methylation level of the Foxp1 promoter, while knockout of circFoxp1 notably reduces this methylation. CircFoxp1 forms an RNA-protein complex with DNMT1, recruiting DNMT1 to the Foxp1 promoter, which leads to promoter hypermethylation and subsequent inactivation, thereby suppressing Foxp1 transcription and enhancing the sensitivity of colorectal cancer cells to the chemotherapeutic agent capecitabine (16). H3K4me3, the trimethylation of lysine 4 on histone H3, is a crucial epigenetic mark used to study gene expression regulation and epigenetic mechanisms (99, 100). In studies of bone marrow-derived mesenchymal stem cells (BMSCs), circFoxp1 has been shown to recruit KDM5B to suppress PTEN expression. CircFoxp1 facilitates the binding of KDM5B to the PTEN promoter, resulting in reduced H3K4me3 occupancy on the PTEN promoter, thereby inhibiting PTEN transcription and promoting elevated expression of RUNX2, OCN, and OPN, which enhances BMSC proliferation and osteogenic differentiation (14). Thus, circFoxp1, through its epigenetic regulation, particularly via DNA methylation and histone H3K4me3 modifications, significantly influences gene expression and cellular behaviors.

#### 4.1.4 Protein translation

Translation, executed by ribosomes, involves initiation, elongation, termination, and ribosomal recycling (101). CircRNA translation can produce novel proteins or isoforms with new physiological functions (102). Additionally, circRNAs with open reading frames (ORFs) can undergo rolling circle amplification in an internal ribosome entry site (IRES)-independent manner, resulting in production rates that are up to one hundred times higher than those of linear transcripts (103). Wang et al. discovered that circFOXP1 inhibits the progression of intrahepatic cholangiocarcinoma (ICC) by encoding a novel protein, circFOXP1-231aa (46) (as shown in **Figure 2F**). Although both circFOXP1 and circFOXP1-231aa are associated with the *FOXP1* gene, circFOXP1-231aa does not affect *FOXP1* gene expression but rather interacts with different molecules through distinct pathways to influence cellular functions. Specifically, circFOXP1-231aa interacts with OTUD4, which regulates the stability of the NCOA4 protein through deubiquitination. This interaction enhances ferroptosis in ICC cells and suppresses ICC recurrence. The efficiency of circRNA translation and its ability to produce novel proteins make it a focal point of research. The study of circFOXP1-231aa highlights how circRNAs, through protein translation, can interact with key molecules to impact cancer progression and recurrence.

#### 4.1.5 PI3K/AKT signaling pathway

The PI3K/AKT signaling pathway is a highly conserved signaling network in eukaryotic cells that promotes cell survival, growth, and cell cycle progression (104). Wu et al. (51) found that circFOXP1 activates the PI3K/AKT/mTOR signaling pathway by regulating the miR-338-3p/ETS1 axis, thereby enhancing the proliferation, invasion, and migration of colorectal cancer cells. Specifically, their research demonstrated that inhibition of miR-338-3p significantly increased the expression levels of p-AKT and p-mTOR proteins, while knockdown of circFOXP1 could reverse this effect. Furthermore, overexpression of ETS1 also elevated the levels of p-AKT and p-mTOR proteins, without significantly affecting the total levels of AKT, mTOR, or β-actin. These findings highlight the crucial role of circFOXP1 in colorectal cancer, particularly in its regulation of the PI3K/AKT/mTOR signaling pathway via the miR-338-3p/ETS1 axis. Additionally, Xin et al. (14) discovered that circFOXP1 activates the PI3K/AKT pathway by suppressing the expression of PTEN. Remedial experiments using insulin-like growth factor 1 (IGF-I) restored the inhibited proliferation and differentiation of BMSCs caused by circFOXP1 knockdown. This indicates that circFOXP1 plays a critical role in regulating BMSC proliferation and differentiation through the PI3K/AKT pathway. Overall, these findings suggest that circFOXP1 exerts extensive biological functions in various diseases through its regulation of the PI3K/AKT signaling pathway.

#### 4.1.6 Canonical and non-canonical Wnt pathways

The Wnt signaling pathways are essential regulators of cellular processes such as growth, differentiation, and migration, and are of significant importance in developmental biology and cancer research (105). These pathways are primarily divided into canonical (or β-catenin-dependent) and non-canonical (or β-catenin-independent) pathways (106). Research has revealed that the expression of circFOXP1 is downregulated upon reprogramming mesenchymal stem cells (MSCs) into human induced pluripotent stem cells (hiPSCs). Concurrently, the RNA levels of WNT5A, ROR2, PIK3CA, and NRAS are notably decreased. Moreover, the expression of genes involved in the non-canonical Wnt pathway, such as the Wnt5a ligand and Fzd1 co-receptor, is suppressed, while mRNA levels of Wnt3a ligand, Lrp6, and Fzd7 receptors, which are components of the canonical Wnt pathway, are significantly upregulated (90). In MSCs, elevated circFOXP1 levels sustain the activity of the non-canonical Wnt pathway, thereby attenuating the function of the canonical Wnt pathway. Conversely, in hiPSCs, the reduced abundance of circFOXP1 allows miR-17-3p and miR-127-5p to mediate the inhibition of the non-canonical Wnt pathway, thereby enhancing the transmission of endogenous canonical Wnt signals. Thus, circFOXP1 plays a pivotal role in modulating Wnt signaling pathways and influencing cellular fate transitions.

### 4.2 Upstream mechanisms

**Figure 4:**
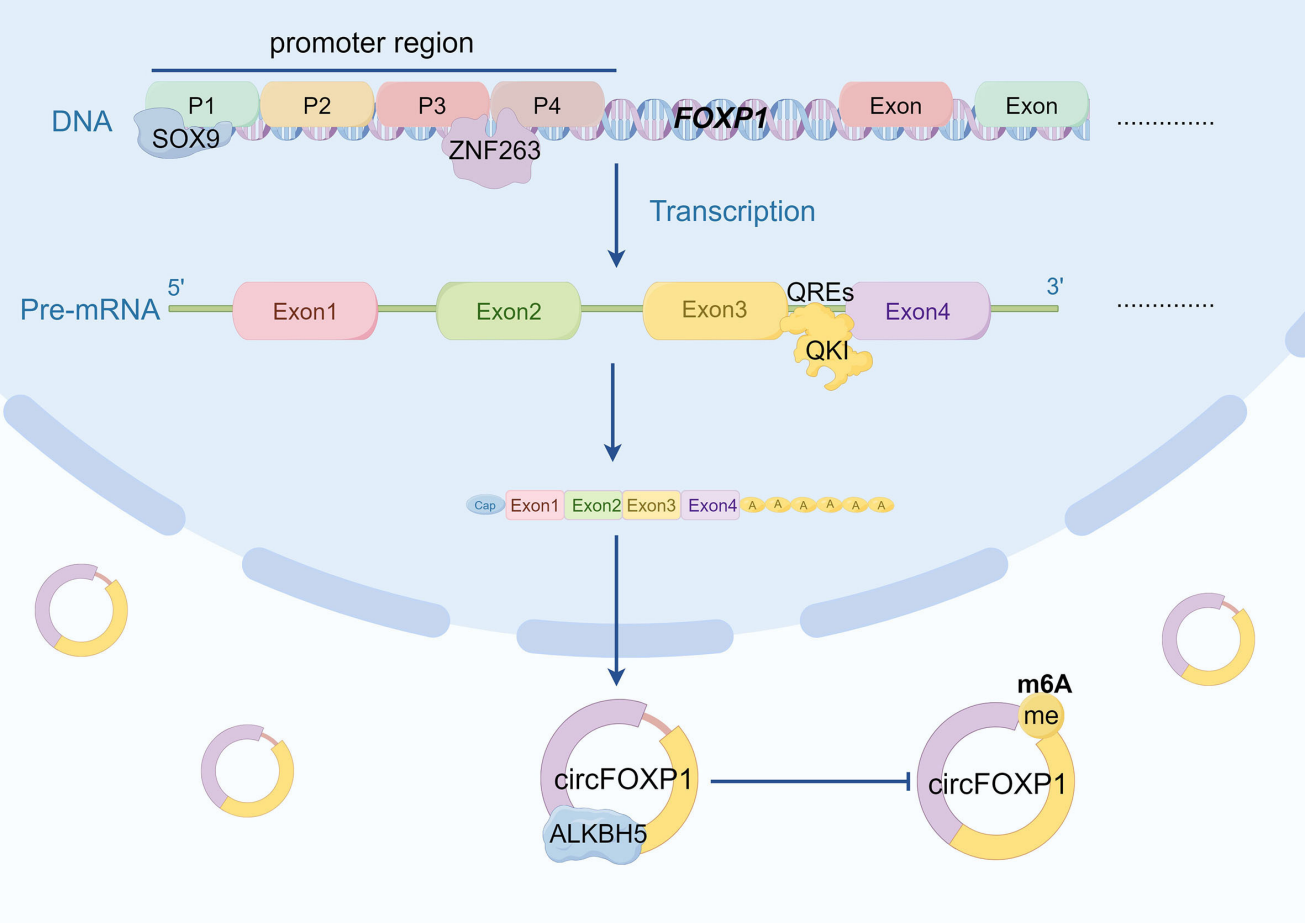
The diagram illustrates the multifaceted regulatory mechanisms governing circFOXP1 expression. Firstly, ZNF263 regulates circFOXP1 expression by binding to the P3 and P4 regions of the FOXP1 promoter. Additionally, ZNFSOX9 modulates circFOXP1 transcription through the P1 binding site. QKI promotes circFOXP1 biogenesis by binding to QREs (QKI response elements). Furthermore, ALKBH5 enhances circFOXP1 expression by reducing m6A modification levels. (by Figdraw2.0).

The functionality and expression of circRNA are influenced not only by its translational processes but also by the intricate regulation of upstream transcriptional and splicing mechanisms (107). Transcription factors, splicing factors, and other regulatory elements modulate the splicing patterns of precursor mRNA, determining the production, stability, and biological roles of circRNA within cells (108). A deeper understanding of these regulatory mechanisms is crucial for elucidating the roles of circRNA in various physiological and pathological contexts. For instance, research has demonstrated that ZNF263 interacts with two splice sites (P3 and P4) on the FOXP1 promoter, thereby regulating the expression of circFOXP1 in renal cell carcinoma (RCC) cells, which promotes RCC cell proliferation, migration, invasion, and the Warburg effect (48). Additionally, in hepatocellular carcinoma (HCC), two SOX9 binding sites have been identified in the promoter region of FOXP1. SOX9 regulates circFOXP1 transcription through the P1 site, leading to increased levels of circFOXP1 in HCC and contributing to tumor progression (47). N6-methyladenosine (m6A) is one of the most common and reversible internal modifications of circRNA (109). Wang et al. discovered that ALKBH5 mediates the regulation of m6A modification of circFOXP1 in gastric cancer (GC). ALKBH5 binds to circFOXP1 in GC cells; overexpression of ALKBH5 decreases the total m6A and m6A levels of circFOXP1, while increasing its expression (43). In addition to transcription factors and epitranscriptomic modifications, circFOXP1 is also regulated by RNA-binding proteins. QKI, a well-known RNA-binding protein, has been reported to promote the formation of circRNA by binding to QKI response elements (QREs) in the exonic flanking introns of circRNAs (110). Studies show that QKI enhances circFOXP1 biogenesis through its interaction with QREs. Under hypoxic conditions, reduced expression of QKI is a primary factor leading to decreased levels of circFOXP1. This reduction in circFOXP1 expression accelerates the ubiquitination and degradation of STAT3 protein, ultimately exacerbating brain injury following cerebral ischemia by activating apoptotic signaling pathways (49). In summary, precise regulation of circFOXP1 expression and function can be achieved through transcription factor control, epitranscriptomic modifications, and RNA-binding protein interactions (**Figure 4**). These mechanisms can modulate disease progression or improve therapeutic outcomes across different disease contexts.

## 5 Potential clinical applications of circFOXP1

### 5.1 Diagnostic biomarker

Globally, cancer incidence approached 20 million new cases in 2022, with nearly 9.7 million cancer-related deaths (52). The rising rates of cancer incidence and mortality continue to make it a leading threat to human health worldwide (111). Investment in cancer prevention can significantly reduce future cancer cases, save lives, and yield economic and social benefits (112). Understanding cancer mechanisms—from basic evolution to optimizing early detection—provides broad prospects for cancer prevention and treatment. Research has demonstrated that circFOXP1 is overexpressed in various tumors, including hepatocellular carcinoma (HCC), gastric cancer (GC), colorectal cancer (CRC), epithelial ovarian cancer (EOC), cutaneous squamous cell carcinoma (cSCC), osteosarcoma, and gallbladder cancer (GBC), compared to normal tissues. Conversely, circFOXP1 expression is reduced in some lung cancers and intrahepatic cholangiocarcinoma (ICC) compared to normal tissues (as shown in **Table 1**). This suggests that circFOXP1 could serve as a histological biomarker for tumor diagnosis. Serological studies are crucial in cancer diagnostics as they provide essential support for early diagnosis, disease monitoring, and treatment assessment by detecting biomarkers in blood (113). For instance, circFOXP1 is significantly overexpressed in the serum of non-small cell lung cancer (NSCLC) patients. Its ROC curve area shows superior diagnostic performance compared to conventional tumor markers such as CEA and CYFRA21-1, indicating better diagnostic advantages (42). Additionally, circFOXP1 levels are significantly elevated in the serum of HCC patients, with an ROC curve area (AUC) of 0.931, suggesting its potential as a promising biomarker for HCC with high diagnostic value (47). In exosome studies, circFOXP1 expression is markedly upregulated in exosomes, and its expression in exosomes from EOC patients with cisplatin resistance is notably higher than in those sensitive to cisplatin (45). Moreover, the potential clinical applications of circFOXP1 extend beyond oncology. Studies have shown that circFOXP1 exhibits abnormal expression in various non-tumor conditions, such as acute ischemic stroke (49), pulmonary fibrosis (17), keloids (50), and atherosclerosis (20). This abnormal expression in diseased tissues suggests that circFOXP1 could serve as a valuable biomarker for these conditions, highlighting its role in diagnostic applications beyond cancer. However, it is essential to note that the evidence supporting these claims is still emerging. Future studies should focus on validating circFOXP1’s utility as a biomarker in these non-tumor contexts through well-designed clinical trials. Additionally, understanding how circFOXP1 might be targeted for therapeutic interventions would significantly enhance its clinical relevance. Current research into circRNA-based therapies shows promise, and circFOXP1 may serve as a novel target for drug development, potentially leading to innovative treatments for both tumor and non-tumor diseases.

The validation of circFOXP1 is critically important due to the unique structure and function of circRNAs. Their stability, closed circular structure, and specific expression patterns make them potential biomarkers, particularly in the diagnosis and treatment of diseases such as cancer. Common techniques, including qRT-PCR, Northern blotting, circRNA overexpression and knockdown, and fluorescence *in situ* hybridization (FISH), can identify and quantify circRNA, ensuring they are not merely mRNA splice variants or other non-coding RNAs (114–117). Each of these methods plays a crucial role in validating circFOXP1 functionality. qRT-PCR boasts high sensitivity and specificity, presenting promising clinical applications, although it requires precise primer design. Northern blotting provides accurate data on RNA size and structure, which is vital in early studies, yet it can be labor-intensive. CircRNA overexpression is beneficial for investigating circRNA’s biological functions, understanding its role in disease processes, and exploring its potential as a therapeutic target. However, overexpression experiments must be meticulously optimized and may not fully replicate *in vivo* conditions. CircRNA knockdown effectively explores circular RNA functions by inhibiting expression and assessing subsequent impacts on cellular processes, thereby identifying circRNAs as therapeutic targets. Nevertheless, the design of siRNAs may pose challenges due to potential off-target effects, and the structural stability of certain circular RNAs complicates effective knockdown. FISH provides valuable insights into circRNA functionality based on cellular distribution, potentially guiding further functional studies. However, compared to other methods, FISH is technically demanding, costly, and may have limited sensitivity. While numerous challenges remain in the validation techniques for circFOXP1, the combined application of these methods significantly enhances our comprehensive understanding of circFOXP1 and its potential applications in disease diagnostics.

### 5.2 Prognostic biomarker

Prognostic biomarkers are instrumental in predicting disease progression and treatment outcomes, assisting physicians in evaluating patient survival expectations, selecting the most appropriate treatment plans, and adjusting therapeutic strategies to optimize patient management and enhance treatment success rates (118). Research on 78 colon cancer patients revealed that circFOXP1 is significantly elevated in stages III-IV and is associated with poorer survival rates. Conversely, Foxp1 levels were significantly lower in these advanced stages, correlating with better survival outcomes, indicating a notable negative correlation between circFOXP1 and Foxp1 expression (16). Interestingly, in 50 patients with lung adenocarcinoma, circFoxp1 expression was significantly associated with tumor size, with high circ FOXP1 expression correlating with better prognosis (38). However, another study on 153 LUAD patients demonstrated that circFOXP1 overexpression was associated with shorter survival compared to lower expression levels, suggesting a discrepancy in its prognostic value (39). Similarly, in 105 non-small cell lung cancer patients, high circFOXP1 expression was significantly associated with advanced T stage and lymph node metastasis, indicating variability in prognostic impact across different patient populations or sample types, necessitating further investigation into its role and mechanisms in lung cancer (42). In 40 gallbladder cancer (GBC) patients, high circFOXP1 levels were closely related to lymph node metastasis and advanced TNM stages (III-IV) (41). Among 56 gastric cancer patients, elevated circFOXP1 expression correlated positively with tumor size, lymph node metastasis, and advanced TNM stages, and was associated with poorer disease-free survival (DFS) and overall survival (OS) (43). In 40 osteosarcoma patients, downregulation of circFOXP1 was negatively associated with OS (44). In 200 cases of epithelial ovarian cancer (EOC), high levels of circFOXP1 in serum exosomes were significantly associated with FIGO stage, primary tumor size, lymph node metastasis, distant metastasis, residual tumor diameter, and clinical response (45). High circFOXP1 expression in 93 HCC patients correlated with larger tumor volume, microvascular invasion, and advanced TNM stages (47). Remarkably, in 200 ICC cases, high circFOXP1 expression predicted better survival and lower recurrence rates, suggesting a potential tumor-suppressive role (46). Overall, circFOXP1’s prognostic value appears to be dependent on cancer type and patient cohort. Further research is needed to elucidate its mechanisms and functions in different tumors for accurate clinical application. Nevertheless, circFOXP1 remains a significant prognostic biomarker in cancer, with its expression levels correlating with tumor stage, grade, OS, microvascular invasion, and clinical response. This highlights the potential of circFOXP1 in assessing cancer malignancy and predicting prognosis, emphasizing the importance of continued development of tumor biomarkers for early diagnosis and recurrence vigilance.

### 5.3 Therapeutic target

In oncology, circFOXP1 is predominantly regarded as an oncogene, driving tumorigenesis in various cancers, including hepatocellular carcinoma and colorectal cancer. Modulating circFOXP1 expression offers the potential for personalized therapeutic strategies aimed at targeting specific molecular alterations within tumors. Therapeutic approaches targeting circFOXP1 include the use of small molecule inhibitors to block its function (119), RNA interference techniques to downregulate its expression (115), CRISPR-Cas13 gene editing to knockout circFOXP1 (120), restoration of miRNA function suppressed by circFOXP1, and the precise delivery of these therapeutic molecules via nanoparticle carriers. Following advances in gene therapy, non-coding RNAs, including circRNAs, have emerged as novel molecular drugs for cancer treatment (121). Research has progressively transitioned circFOXP1 from a molecular object of basic studies to a candidate drug for clinical applications, though it is still primarily in the preclinical research stage. As a molecular targeted drug, circFOXP1 represents a new paradigm in tumor therapy and advances precision medicine. CircFOXP1 also holds substantial therapeutic potential beyond oncology. Its unique expression characteristics and regulatory roles make it a crucial target for developing new therapeutic strategies for various non-cancerous diseases. For instance, in acute ischemic stroke, circFOXP1 can inhibit cell apoptosis by downregulating Bax and caspase-3, and upregulating Bcl2, thereby reducing brain infarction area under hypoxic conditions (49). Moreover, in pulmonary fibrosis, treating with hucMSCs has shown that downregulation of circFOXP1 results in thinner alveolar walls in mice, effectively improving alveolar structure, significantly reducing alveolar inflammation, and decreasing collagen deposition, including fibrotic proteins such as vimentin, α-SMA, collagen I and III, and differentiation-associated protein S100 calcium-binding protein A4 (17). Additionally, in benign tumors such as keloids, knocking down circFOXP1 reduces cell proliferation and migration, increases apoptosis, and mitigates cellular inflammation and oxidative stress (50). These findings suggest that modulating circFOXP1 expression could be therapeutically beneficial for a range of diseases, highlighting its potential significance in research and treatment of various clinical conditions. However, clinical treatment strategies based on circFOXP1-targeted drugs have not yet seen widespread application. Future research should focus on evaluating the safety, stability, and efficacy of circFOXP1-targeted drugs through large-scale randomized clinical trials.

Moreover, recent studies on the therapeutic role of circFOXP1 in tumors have revealed that lung cancer cells do not express circFOXP1 prior to chemotherapy, but its presence can be detected post-treatment, with expression levels further elevated following combination therapy (15). This observation suggests that circFOXP1 may play a dynamic role in cellular responses to chemotherapy, potentially serving as a compensatory mechanism following treatment. Such dynamic changes position circFOXP1 as a potential diagnostic marker or a tracking marker for disease progression, particularly in evaluating chemotherapy efficacy. In colorectal cancer cells, the knockdown of circFOXP1 significantly enhances sensitivity to capecitabine, both *in vitro* and *in vivo (* 16). This increased drug sensitivity is mediated by the regulation of Foxp1 DNA methylation and demethylation, indicating that circFOXP1 plays a pivotal role in the epigenetic regulation of chemotherapy resistance. By modulating circFOXP1, it may be possible to enhance patient sensitivity to capecitabine, thereby increasing the likelihood of successful treatment outcomes. Furthermore, in ovarian cancer, circFOXP1 regulates miR-22 and miR-150-3p through a sponge mechanism, positively influencing the expression of CEBPG and FMNL3, thereby promoting both cancer cell proliferation and enhancing the sensitivity of epithelial ovarian cancer cells to cisplatin (45). Restoring the functions of miR-22 and miR-150-3p by alleviating circFOXP1’s suppression could reinstate their regulatory effects on tumor suppressor genes, thereby exerting anti-tumor activity. CircFOXP1 may act as a key regulator of chemoresistance across various cancer types, demonstrating substantial potential as a therapeutic target through multiple avenues, including drug targeting, gene engineering, and restoration of miRNA functions.

## 6 Conclusion and future perspectives

circFOXP1, as a circular RNA, plays a pivotal role in the progression of human diseases. This molecule exhibits unique biological functions and participates in the pathogenesis of various conditions through diverse molecular mechanisms. In the context of oncology, the expression of circFOXP1 within tumor tissues influences critical factors such as tumor volume, TNM staging, lymph node metastasis, tumor classification, overall survival rates, microvascular invasion, and clinical response. Conversely, in non-cancerous studies, circFOXP1 expression impacts ischemic stroke patients by affecting infarct size, adverse prognosis, fibrinogen and D-dimer levels, and the incidence of recurrent pregnancy loss. circFOXP1 functions as a molecular sponge for a range of microRNAs, including miR-127-5p, miR-338-3p, miR-547-5p, miR-490-3p, miR-520-5p, miR-370, miR-185-5p, miR-18a-5p, miR-22, miR-150-3p, miR-875-3p, miR-421, miR-423-5p, miR-143-3p, and miR-33a-5p, binding specifically to these miRNAs. By competing with these miRNAs for downstream targets, such as ADCY7, ETS1, RND3, SYT1, SLC7A11, WNT1, PKLR, SOX4, CDKN2AIP, CEBPG, FMNL3, U2AF2, S100A11, BCL-2, and FOXP1, circFOXP1 modulates disease progression through intricate circRNA–miRNA–mRNA networks. Additionally, circFOXP1 interacts with RNA-binding proteins and regulates protein translation, further influencing human disease outcomes. It also targets specific signaling pathways, including both canonical and non-canonical Wnt pathways, as well as the PI3K/AKT pathway. Through its involvement in processes such as cell proliferation, migration, invasion, drug resistance, the Warburg effect, and EMT, circFOXP1 emerges as a promising candidate for diagnostic and therapeutic applications in human diseases.

The utilization of circFOXP1 as a biomarker presents distinct advantages, primarily attributable to its relatively high stability, allowing it to persist in plasma and other biological samples over extended periods; this characteristic renders circFOXP1 an ideal candidate for early diagnosis and monitoring of disease progression. Moreover, the expression levels of circFOXP1 within the tumor microenvironment exhibit significant correlations with various clinical parameters, such as tumor volume, lymph node metastasis, and patient survival, thereby underscoring its critical role in tumorigenesis and progression. However, it is noteworthy that the expression levels of circFOXP1 may vary markedly across different tumor types; for instance, in cholangiocarcinoma (46), circFOXP1 expression is typically lower than that in normal tissues, whereas conflicting results have been reported regarding its expression in certain lung cancers (38), indicating discrepancies among different studies. Such variability in expression may limit the applicability of circFOXP1 as a universal biomarker; consequently, caution is warranted when utilizing circFOXP1 in various cancer types and clinical contexts. Given the multifaceted roles of circFOXP1 in tumors, we propose its consideration as a potential diagnostic and prognostic biomarker, and further research into the specific roles of circFOXP1 across different cancers could provide a theoretical foundation for its application in personalized therapy. For instance, circFOXP1 can compete with specific microRNAs, thereby influencing the expression of downstream genes and presenting new opportunities for targeted therapies; additionally, circFOXP1’s interactions with RNA-binding proteins, along with its regulatory effects on cellular proliferation, migration, and invasion, further validate its potential as a therapeutic marker. Thus, a comprehensive exploration of circFOXP1’s mechanisms in tumor progression is essential, not only to enhance our understanding of its biological functions but also to establish a basis for its translational applications in clinical settings. Future research should focus on the identification and characterization of different circFOXP1 isoforms to facilitate a more thorough understanding of its potential applications across various diseases.

Despite recent advances in understanding the role of circFOXP1 in human diseases, several critical questions remain unresolved, and its clinical applications and mechanisms of action warrant further investigation. Initially, while examining the expression of circFOXP1 across various cancers, significant differences in expression patterns were noted. For instance, circFOXP1 is generally expressed at lower levels in cholangiocarcinoma compared to normal tissues. This observation suggests that circFOXP1 may play a distinct role in the pathogenesis of ICC relative to other cancer types. However, the expression levels and specific functions of circFOXP1 in lung cancer remain contentious, with existing studies providing conflicting results. The precise role and biological functions of circFOXP1 in lung cancer are still not fully elucidated and necessitate further detailed exploration. These discrepancies in expression and functional roles underscore the importance of considering the context-specific role of circFOXP1 in cancer research, offering valuable insights for future investigations. Furthermore, the *FOXP1* gene encodes various circFOXP1 isoforms, highlighting the need for comprehensive identification and characterization of all circFOXP1 variants. Future research should involve larger and more diverse populations to better understand the characteristics of circFOXP1. Additionally, integrating circFOXP1 into personalized treatment regimens requires a deeper exploration of its precise mechanisms in disease progression. Finally, the establishment of standardized nomenclature for circRNAs is crucial for the systematic organization and consistency in research.

This review elucidates the latest advancements in research on circFOXP1, emphasizing its clinical significance and biological functions across a spectrum of human diseases. As a potential immune checkpoint molecule, circFOXP1 exerts crucial biological effects within cells through mechanisms such as ceRNA networks, interactions with RNA-binding proteins, and modulation of protein translation. Future research may position circFOXP1 as an innovative biomarker, offering novel targets for the diagnosis and treatment of human diseases.

## Glossary

circRNA: Circular RNA
circFOXP1: Circular RNA Forkhead Box P1
Wnt: Wingless-Related Integration Site
PI3K/AKT: Phosphoinositide 3-Kinase/Protein Kinase B
FOXP1: Forkhead Box P1
FOX: Forkhead Box
EcircRNA: Exonic Circular RNA
ciRNA: Circular Intron RNA
EIciRNA: Exon-Intron Circular RNA
OS: Overall Survival
DFS: Disease-free Survival
CRC: Colorectal Cancer
SCLC: Small Cell Lung Cancer
NSCLC: Non-Small Cell Lung Cancer
LUAD: Lung Adenocarcinoma
GC: Gastric Cancer
HCC: Hepatocellular Carcinoma
CTNNB1: Catenin Beta 1
ROC: Receiver Operating Characteristic
ICC: Intrahepatic Cholangiocarcinoma
GBC: Gallbladder Cancer
PCNA: Proliferating Cell Nuclear Antigen
MMP9: Matrix Metallopeptidase 9
EOC: Epithelial Ovarian Cancer
DDP: Cisplatin
RCC: Renal Cell Carcinoma
cSCC: Cutaneous Squamous Cell Carcinoma
ONFH: Osteonecrosis of the Femoral Head
BMSC: Bone Marrow Mesenchymal Stem Cell
RUNX2: Runt-Related Transcription Factor 2
OPN: Osteopontin
OCN: Osteocalcin
AIS: Acute Ischemic Stroke
PF: Pulmonary Fibrosis
hucMSC: Human Umbilical Cord Mesenchymal Stem Cell
α-SMA: Alpha-Smooth Muscle Actin
S100A4: S100 Calcium Binding Protein A4
RPL: Recurrent Pregnancy Loss
HSF: Human Skin Fibroblasts
ECM: Extracellular Matrix
AS: Atherosclerosis
HFD: High-fat Diet
IL-6: Interleukin 6
TNF-α: Tumor Necrosis Factor Alpha
IL-1β: Interleukin 1 Beta
OP: Osteoporosis
MSC: Mesenchymal Stem Cell
PDPN: Podoplanin
GLI1: GLI Family Zinc Finger 1
ADCY7: Adenylate Cyclase 7
ETS1: E26 Transformation Specific-1
mTOR: Mechanistic Target of Rapamycin
DNMT1: DNA (Cytosine-5)-Methyltransferase 1
RND3: Rho Family GTPase 3
SYT1: Synaptotagmin 1
SLC7A11: Solute Carrier Family 7 Member 11
WNT1: Wnt Family Member 1
PTBP1: Polypyrimidine Tract Binding Protein 1
PKLR: Pyruvate Kinase L/R
ALKBH5: AlkB Homolog 5, RNA Demethylase
SOX4: SRY-Box Transcription Factor 4
CDKN2AIP: Cyclin Dependent Kinase Inhibitor 2A Interacting Protein
FMNL3: Formin Like 3
OTUD4: OTU Deubiquitinase 4
NCOA4: Nuclear Receptor Coactivator 4
SOX9: SRY-Box Transcription Factor 9
ZNF263: Zinc Finger Protein 263
KDM5B: Lysine Demethylase 5B
PTEN: Phosphatase And Tensin Homolog
QKI: Quaking (RNA Binding Protein)
STAT3: Signal Transducer and Activator of Transcription 3
EZH2: Enhancer of Zeste Homolog 2
STAT1: Signal Transducer and Activator of Transcription 1
RACK1: Receptor for Activated C Kinase 1
BCL-2: B-Cell Lymphoma 2
MRE: MicroRNA Response Element
EMT: Epithelial-Mesenchymal Transition
HuR: Human Antigen R
WNT5A: Wnt Family Member 5A
ROR2: Receptor Tyrosine Kinase-Like Orphan Receptor 2
NRAS: Neuroblastoma RAS Viral Oncogene Homolog
Lrp6: Low-Density Lipoprotein Receptor-Related Protein 6
Fzd7: Frizzled Class Receptor 7
QRE: QKI Response Element
AUC: Area Under the Curve
